# The landscape of antimicrobial resistance in the neonatal and multi-host pathogen group B *Streptococcus:* review from a One Health perspective

**DOI:** 10.3389/fmicb.2022.943413

**Published:** 2022-07-28

**Authors:** Laura M. A. Oliveira, Leandro C. Simões, Natalia S. Costa, Ruth N. Zadoks, Tatiana C. A. Pinto

**Affiliations:** ^1^Departamento de Microbiologia Médica, Instituto de Microbiologia Paulo de Góes, Universidade Federal do Rio de Janeiro, Rio de Janeiro, RJ, Brazil; ^2^Sydney School of Veterinary Science, Faculty of Science, University of Sydney, Camden, NSW, Australia

**Keywords:** group B *Streptococcus*, antimicrobial resistance, One Health, pediatric bacterial infection, COVID-19

## Abstract

Group B *Streptococcus* (GBS) stands out as a major agent of pediatric disease in humans, being responsible for 392,000 invasive disease cases and 91,000 deaths in infants each year across the world. Moreover, GBS, also known as *Streptococcus agalactiae*, is an important agent of infections in animal hosts, notably cattle and fish. GBS population structure is composed of multiple clades that differ in virulence, antimicrobial resistance (AMR), and niche adaptation; however, there is growing evidence of interspecies transmission, both from evolutionary analysis and from disease investigations. The prevention of GBS infections through vaccination is desirable in humans as well as animals because it reduces the burden of GBS disease and reduces our reliance on antimicrobials, and the risk of adverse reactions or selection for AMR. In this perspective article, we navigate through the landscape of AMR in the pediatric and multi-host pathogen GBS under the One Health perspective and discuss the use of antimicrobials to control GBS disease, the evolution of AMR in the GBS population, and the future perspectives of resistant GBS infections in the post-pandemic era.

## Introduction

Group B *Streptococcus* (GBS) is a recognized agent of perinatal infections in humans (Raabe and Shane, 2019). Human neonatal invasive disease can present as meningitis, sepsis, and bacteremic pneumonia, and accounts for 392,000 cases, 91,000 deaths, and 37,000 cases of survivor children with neurodevelopmental impairment globally each year (World Health Organization, 2021). GBS is composed of several lineages, for example as defined based on multi-locus sequence typing and clonal complexes or on BAPS clustering, that may differ in their virulence traits, AMR profile, and host adaptation (Botelho et al., 2018a; Richards et al., 2019).

In the medical literature, host adaptation is mostly considered in the context of host age, i.e., pediatric vs. adult GBS (Raabe and Shane, 2019; Watkins et al., 2019), but GBS is also a cause of infectious diseases among food-producing animals, including cattle (Pinto et al., 2013; Cobo-Angel et al., 2019), fishes (Delannoy et al., 2013; Leal et al., 2019) and camels (Fischer et al., 2013; Seligsohn et al., 2021b) and it has been detected in companion animals (Maeda et al., 2022), laboratory animals (Bodi Winn et al., 2018), and wildlife (Bowater et al., 2012). There is evidence for GBS transmission between humans and animals from evolutionary (Barkham et al., 2019; Richards et al., 2019; Crestani et al., 2021) as well as epidemiological studies (Kalimuddin et al., 2017; Sørensen et al., 2019; Seligsohn et al., 2021a) with transmission *via* multiple routes and in both directions (Botelho et al., 2018a; Crestani et al., 2021). The frequency of such transmission events, whether through direct contact, food, or the environment, is poorly known (Botelho et al., 2018a). In addition to risks associated with being a versatile multi-host pathogen, clindamycin and erythromycin resistance in GBS have been highlighted as a “concerning threat” in terms of antimicrobial resistance (AMR; Centers for Disease Control and Prevention, 2019). Indeed, the two may be interrelated as different hosts are exposed to different antimicrobial use pressures, and selection for AMR in one host may be followed by host-species jumping of lineages, amplification, and onward transmission in another host (Richards et al., 2019; Crestani et al., 2021). Thus, to fully understand the epidemiology and control of GBS in humans and animals, a One Health approach is needed, i.e., a collaborative, multisectoral and transdisciplinary approach that acts at the local, regional, national, and global levels to promote the health of people, animals, and their shared environment. The One Health approach can be applied to prevent outbreaks of zoonotic diseases in animals and humans, improve food safety and security, reduce AMR in bacterial pathogens, and promote human and animal health (Hernando-Amado et al., 2020).

The increasing prevalence of AMR in bacterial pathogens is a global concern because it impacts on human and animal health and the environment. AMR is considered a priority topic by international organizations such as the World Health Organization and World Organization for Animal Health [OIE (World Organisation Animal Health), 2021]. The importance of AMR emergence and the One Health approach have been reinforced during the COVID-19 pandemic. Personal and behavioral measures implemented to mitigate the pandemic, like social distancing and hand hygiene, may reduce the transmission of healthcare-associated pathogens (Brueggemann et al., 2021). However, antimicrobial agents (including the broad-spectrum ones) were widely prescribed in healthcare systems to treat COVID-19 patients, which may have contributed to selection for AMR (Rawson et al., 2020; Garcia-Vidal et al., 2021; Westblade et al., 2021). In addition, the resulting antimicrobial residues and resistance genes would have been eliminated with fecal bacteria *via* hospital sewage into the environment, which could lead to contamination of drinking water, soil, animals, and other ecological niches (McEwen and Collignon, 2018). Antimicrobial misuse and overuse drives resistance selection and can contribute to the clonal spread of drug-resistant pathogens across animal and human hosts and the horizontal transmission of resistance determinants (McEwen and Collignon, 2018). Lateral gene transfer mediated by mobile genetic elements is amongst the major forces that drive GBS genome plasticity and evolution (Pinto et al., 2014; Chen, 2019).

Although the short-and long-term impacts of COVID-19 on AMR and drug-resistant infections are still largely speculative, we discuss what is currently known about the pediatric and multi-host pathogen GBS through an AMR and One Health lens, and how the COVID-19 pandemic might impact its evolution.

## Use of antimicrobials to control GBS disease and the panorama of antimicrobial resistance

Prevention of GBS disease in neonates and pregnant women relies on the use of antimicrobial agents, since a human GBS vaccine has not been licensed yet (Verani et al., 2010; Le Doare et al., 2017). Indeed, the evolution of AMR in GBS appears to be driven by selection pressure in the human GBS population (Da Cunha et al., 2014; Richards et al., 2019). GBS is one of the main agents of bovine mastitis, which causes major economic losses to the dairy industry (FAO, 2014). GBS bovine mastitis has largely been eradicated in certain high-income countries after the implementation of monitoring programs and guidelines for good biosecurity practices on the farms (Jørgensen et al., 2016; Crestani et al., 2021). However, in many low-and middle-income countries and emerging economies, including those top-ranked in the global milk industry like Brazil and China, adoption of good prevention and control practices faces several barriers and GBS prevalence remains high in dairy herds [Botelho et al., 2018a; OIE (World Organisation Animal Health), 2021]. This may lead to frequent use of antimicrobials without proper technical advice, including unnecessary use of broad-spectrum antimicrobials when GBS is generally susceptible to penicillin. Such excessive use of antimicrobials in food-producing animals, including highest-priority critically important antimicrobials such as 3rd or 4th generation cephalosporins, contributes to selection for AMR in multi-host pathogens and can limit the therapeutic options for treatment of infections in animals and humans (Gomes and Henriques, 2016; Ruegg, 2017). In the 1980s, Berghash and Dunny already reported GBS strains resistant to beta-lactams, tetracyclines and aminoglycosides isolated from cattle subjected to routine antibiotic exposure in dairy herds in the US (Berghash and Dunny, 1985). The situation is complex, however, as AMR prevalence in bovine GBS is generally low in the US (Erskine et al., 2002), and less common than in human isolates from the same region (Dogan et al., 2005), whereas the opposite is true in, for example, Brazil (Pinto et al., 2013).

Intrapartum antibiotic prophylaxis (IAP), administered based on results of pre-partum bacteriological screening or peri-partum risk factors, is the recommended preventative approach for early-onset GBS disease in newborns and is based on the administration of penicillin or ampicillin to GBS-colonized women during labor. These guidelines were first published in 1996 and were recently updated (American Academy of Pediatrics, 2020); however, the first report of intrapartum antibiotic administration as a preventive measure against neonatal GBS disease dates from 1973 (Franciosi et al., 1973). In the case of patients allergic to penicillin, alternatives including cefazolin, clindamycin, or vancomycin are recommended for IAP. IAP has been highly effective in preventing the occurrence of early-onset GBS disease and has saved the lives of thousands of newborns across the world; however, it does not protect against other forms of GBS disease and imposes the risk of AMR selective pressure. In addition, it may impact on the infant microbiome (Garcia, 2021). The ideal preventive measure against human GBS disease would be a maternal vaccine. There are promising approaches at clinical trials, and it is expected that by 2026 at least one GBS vaccine will be licensed for maternal immunization (World Health Organization, 2021).

Non-susceptibility of human GBS to beta-lactams was first reported from Japan in 1995 in a strain belonging to serotype III and recovered from the sputum of an adult patient. This strain was non-susceptible to penicillin, oxacillin, and cephalosporins and presented point mutations in *pbp* genes (Kimura et al., 2008). Since then, the identification of beta-lactam non-susceptible GBS isolates in humans remains sporadic and mostly associated with point mutations in *pbp* genes (Gaudreau et al., 2010; Longtin et al., 2011; Nagano et al., 2012; Metcalf et al., 2017; Yi et al., 2019). In the US, 0.5% of GBS isolates recovered from non-pregnant adults between 2008 and 2016 were non-susceptible to at least one beta-lactam antibiotic tested, and among them, nearly half harbored mutations in the *pbp*2x gene (Watkins et al., 2019). Invasive human GBS strains with reduced penicillin susceptibility, additional AMR markers and major virulence genes are starting to emerge (Koide et al., 2022).

To date, most animal GBS are penicillin-susceptible based on studies performed in Asia, North, Central and Latin America, and Europe (Nam et al., 2009; Lindeman et al., 2013; Soto et al., 2015; El Garch et al., 2020; Hernandez et al., 2021; Lin et al., 2021). As an exception to this rule, Hu et al. (2018) studied 129 GBS isolates from mastitic cows on 16 farms in three provinces of Central and Northeast China and found that they were all non-susceptible to penicillin G and had mutations in *pbp*1B, *pbp*2B and *pbp*2X genes.

Resistance of GBS to lincosamide and macrolide antibiotics is more common, and the prevalence of resistance to clindamycin and erythromycin have markedly increased in recent years, and nearly half of human GBS isolates in the US are macrolide resistant (Centers for Disease Control and Prevention, 2019). Thus, the use of clindamycin during IAP is now conditional on antimicrobial susceptibility testing results. Nanduri et al. (2019) showed an increasing trend of erythromycin resistant GBS isolates recovered from neonatal disease in the US between 2006 (34.7%) and 2015 (49.1%), which may be a consequence of the wide implementation of IAP in the country. Elsewhere, the prevalence of macrolide or lincosamide resistance ranges from 1.9 to 36%, and resistance is mainly associated with the *erm*B and *mef* genes (Pinto et al., 2013; Teatero et al., 2017; Botelho et al., 2018b; Watkins et al., 2019). In dairy cattle, the prevalence of resistance to erythromycin and clindamycin varies from 1 to 30% and 3 to 40%, respectively, with large differences between countries and studies (Denamiel et al., 2005; Dogan et al., 2005; Kalmus et al., 2011; Pinto et al., 2013; Rato et al., 2013; Entorf et al., 2016; Tomazi et al., 2018).

Although less common, GBS isolates resistant to aminoglycosides, fluoroquinolones and vancomycin have also been reported (Dogan et al., 2005; Nam et al., 2009; Pinto et al., 2013; Rato et al., 2013; Srinivasan et al., 2014; Hays et al., 2016; Hawkins et al., 2017; Botelho et al., 2018b; Watkins et al., 2019). In France, Hays et al. (2016) reported a rise in the prevalence of high-level aminoglycoside resistant GBS isolates recovered from human sources, from 6.4% in 2007 to 8.8% in 2014, which harbored the *aac(6′)Ie-aph(2″)Ia* gene. Resistance to fluoroquinolones has been reported sporadically in GBS from humans and companion animals (dogs, cats) and occurs mainly due to point mutations in specific regions of *gyr*A and *par*C genes (Wehbeh et al., 2005; Hays et al., 2016; Botelho et al., 2018b; Watkins et al., 2019; Maeda et al., 2022), whereby infection of GBS in companion animals is usually attributed to spill-over from humans. Piccinelli et al. (2015) reported the emergence of levofloxacin resistant GBS isolates belonging to the clonal complex (CC) 19 and recovered from pregnant women in Italy. Vancomycin resistance was detected in two invasive GBS isolates of serotype II and sequence type (ST) 22 recovered from adult patients with sepsis and occurred due to the acquisition of a *van*G operon, which was probably acquired from an *E. faecalis* donor (Srinivasan et al., 2014).

## Evolution of antimicrobial resistance in GBS

The One Health approach highlights the interplay between people, animals, and our environment and establishes strategies to overcome health issues that arise from the imbalance at the human-animal-environmental interface, including zoonotic diseases and AMR (Hernando-Amado et al., 2020). In this context, multi-species surveillance of GBS molecular and cellular characteristics is crucial for the design of vaccines, detection of transmission modes and reservoirs and tracking of antimicrobial resistant and virulent GBS strains.

The GBS population is classified in several lineages that can be differentiated according to niche adaptation, virulence potential, and genetic relatedness. For example, ST17 is a hypervirulent lineage associated with meningitis in infants; CC61 is a bovine-adapted lineage known for causing mastitis in dairy cattle; CC552 represents a lineage that is unique to coldblooded species (fish and frogs); and CC616 is limited to camels (Delannoy et al., 2013; Gori et al., 2020; Crestani et al., 2021; Seligsohn et al., 2021b). In addition to such host-adapted lineages, the species includes host-generalists, such as CC10, which includes GBS of human, dog, rat, cattle, dolphin, fish, and frog origin (Delannoy et al., 2013; Da Cunha et al., 2014; Leal et al., 2019; Crestani et al., 2021; Maeda et al., 2022). A member of this clade, ST283, caused a major outbreak of human GBS disease in Singapore, which was traced to consumption of contaminated fish (Kalimuddin et al., 2017). Exploration of contemporaneous and collocated GBS isolates from people and cattle also supports the possibility of inter-species transmission, with study designs including on-farm studies (Cobo-Angel et al., 2019; Sørensen et al., 2019) and population level studies (Lyhs et al., 2016). Even in camels, the first evidence of potential human-to-animal transmission has recently been detected (Seligsohn et al., 2021a). Using evolutionary evidence rather than data from contemporaneous and collocated human and animal isolates, Crestani et al. (2021) evaluated a large set of historical and contemporary bovine GBS isolates and showed that CC61 was replaced in dairy cattle in Sweden by new lineages harboring the *tet*M resistance marker, suggesting reverse zoonotic transmission of human lineages after successful elimination of the bovine-adapted lineage. Evolutionary analysis conducted on a large multi-host dataset shows that human-to-cattle transmission is more likely than cattle-to-human transmission, whereas the human-to-fish and fish-to-human migration events are evenly balanced (Richards et al., 2019). GBS lineages adapted to their hosts, whether humans or other animals, can follow evolutionary paths that ultimately allow the colonization and adaptation to a new host (Richards et al., 2019), and AMR may play a role in this process by driving the clonal expansion of successful lineages (Da Cunha et al., 2014; Chen, 2019).

Data on AMR evolution in GBS has been derived from large-scale monitoring programs and epidemiological studies performed across the world. Such studies allow tracking of the emergence of new resistance phenotypes and genotypes and of resistance trends over time [Metcalf et al., 2017; OIE (World Organisation Animal Health), 2021]. The advancement of whole genome sequencing has broadened our knowledge and power to investigate resistance determinants and mechanisms behind the phenotype and may even replace conventional phenotypic testing in the future (Metcalf et al., 2017).

The emergence and spread of AMR may have occurred independently among GBS from human and animal hosts and has mostly been driven by a combination of horizontal gene transfer and clonal expansion (Pinto et al., 2013; Chen, 2019). However, AMR can also be acquired by point mutations in specific genes followed by clonal expansion, as is the case for resistance to beta-lactams (*pbp* genes) and fluoroquinolones (*gyr*A and *par*C genes; Metcalf et al., 2017). It has been suggested that acquisition of the *tet*M gene, which occurred as early as 1917 for CC17, i.e., well before the industrialization of animal agriculture or the use of antimicrobial agents in farming, was a crucial step in GBS evolution, as it allowed the emergence of lineages associated with human perinatal infections during the 1960s in the face of selective pressure imposed by the wide use of tetracyclines in human medicine. The *tet*M gene is usually carried by conjugative transposons of the Tn*916* and *Tn*5801 families and is the predominant tetracycline resistance determinant in human GBS (Da Cunha et al., 2014), whereas tetracycline resistance in bovine GBS may be due to *tet*M or *tet*O (Dogan et al., 2005; Crestani et al., 2021). Da Cunha et al. (2014) suggested that acquisition of tetracycline resistance led to the simultaneous selection and spread of virulent GBS strains, like the CC17 lineage, even though tetracycline is not commonly used for GBS treatment. Subsequently, the acquisition of determinants conferring resistance to erythromycin and clindamycin was considered important for the emergence of GBS lineages associated with infections among non-pregnant adults during the 1990s (Morales et al., 1999; Hayes et al., 2020).

Recent studies show that the prevalence of tetracycline resistance ranges from 80 to 100% among isolates from pregnant women, neonates, and non-pregnant adults (Teatero et al., 2017; Botelho et al., 2018b; Watkins et al., 2019). The prevalence of tetracycline resistance is lower among bovine isolates of GBS strains, ranging from 14 to 60% depending on the country (Brown and Roberts, 1991; Dogan et al., 2005; Nam et al., 2009; Rato et al., 2013), consistent with the idea that tetracycline resistance is a marker of human host-adaptation, with spillover into the bovine population (Richards et al., 2019; Crestani et al., 2021). In fish, tetracycline resistance profiles have primarily been studied in the multi-host lineage ST283, showing striking differences over time and between countries that could not easily be linked to antimicrobial use (Barkham et al., 2019). Considering the widespread use of antimicrobials in aquaculture, and reports of multidrug resistance in a GBS isolate from tilapia in Japan (Li et al., 2020), further monitoring of AMR in terrestrial as well as aquatic GBS is needed.

## Discussion and future perspectives

GBS is a versatile multi-host pathogen with host-specialist and host-generalist strains. Although tetracycline resistance is primarily associated with human GBS, AMR may also confer survival advantages in other host populations that are exposed to selective pressure from antimicrobial use. AMR alone is not sufficient for “host jumping” as adaptation of GBS to the bovine host relies on the presence of a lactose operon rather than the presence of AMR (Lyhs et al., 2016; Richards et al., 2019). As GBS genomic surveillance evolves and expands, we will also expand our ability to identify new resistance mechanisms and elucidate evolutionary pathways that can explain the success of GBS in the One Health context. Meanwhile, the main challenges remain to tackle AMR spread and decrease the burden of GBS neonatal and maternal disease by making vaccines available, first and foremost for humans, but possibly also for major food-producing animal species. It is expected that the wide implementation of a maternal GBS vaccine would help to slow down AMR emergence, but it should be considered that this will happen in a post-pandemic era. Although COVID-19 is a viral disease, the pandemic has led to an increased and indiscriminate use of antimicrobials, including broad-spectrum antibacterials, in healthcare systems in many parts of the world (Daria and Islam, 2022; Friedli et al., 2022). Additionally, increased antimicrobial use during COVID-19 has been documented to lead to increased AMR, with the potential to have long-term consequences for AMR (Meschiari et al., 2022). It is unknown whether this selective pressure has affected the composition of the GBS population, either by selecting for AMR in multiple clades or by conferring a survival advantage to specific clades with pre-existing AMR, as observed when GBS first emerged as a human pathogen. If such a shift in population composition were to occur, this could potentially affect the extent to which current vaccine candidates represent the global human GBS population. Leading GBS vaccine candidates are supposed to be licensed for maternal use by 2026 and vaccine effects on curbing AMR and drug-resistant infections will be seen afterwards (World Health Organization, 2021). Hence, we postulate that COVID-19 might impact on GBS disease control through short-term and long-term effects on the efficacy of antimicrobials and vaccines, respectively. Ongoing tracking of the evolution of AMR in GBS from humans and animals is, therefore, crucial to better assess the impact of vaccines and other prophylactic measures designed in the pre-pandemic world. As the past has shown, GBS is a versatile pathogen able to circumvent hurdles to thrive in the present and to rewrite the future.

## Data availability statement

The original contributions presented in the study are included in the article/supplementary material, further inquiries can be directed to the corresponding author.

## Author contributions

LO, RZ, and TP developed the concept of the manuscript. LO and TP wrote the initial draft. LO, LS, NC, RZ, and TP have critically read, advised on improvements concerning the science and general outline of the manuscript. All authors contributed to the article and approved the submitted version.

## Funding

This work was supported by the International Veterinary Vaccinology Network (IVVN) Fellowship Programme awarded to LO under mentorship of TP and RZ.

## Conflict of interest

The authors declare that the research was conducted in the absence of any commercial or financial relationships that could be construed as a potential conflict of interest.

## Publisher’s note

All claims expressed in this article are solely those of the authors and do not necessarily represent those of their affiliated organizations, or those of the publisher, the editors and the reviewers. Any product that may be evaluated in this article, or claim that may be made by its manufacturer, is not guaranteed or endorsed by the publisher.
